# Supplementary material for: Osa-miR11117 Targets OsPAO4 to Regulate Rice Immunity against the Blast Fungus Magnaporthe oryzae
Source: Int J Mol Sci. 2023 Nov 7;24(22):16052. doi: 10.3390/ijms242216052 (PMC10670930; doi:10.3390/ijms242216052)
Supplement: Supplementary file 1 [file ijms-24-16052-s001.zip › ijms-2683114 - Figures S1-S4.pdf]

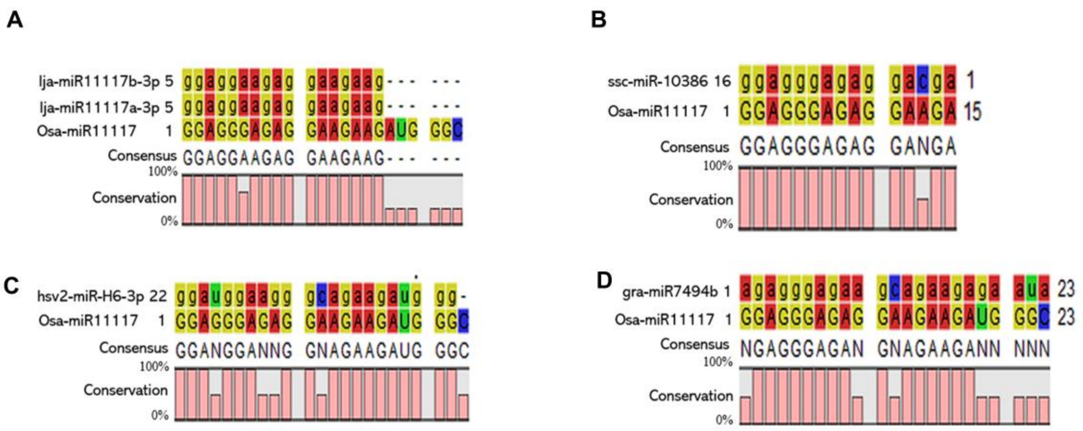

Fig. S1 Comparative homology analysis of osa-miR11117 with partial miRNAs from other species. (A) The senquence of Osa-miR11117 was aligned with the miRNA sequences of lja-miR11117a-3p/lja-miR11117b-3p from lotus, (B) ssc-mir-10386 from *Sus scrofa*, (C) hsv2-miR-H6-3p from herpesvirus, (D) gra-miR7494b from canary.

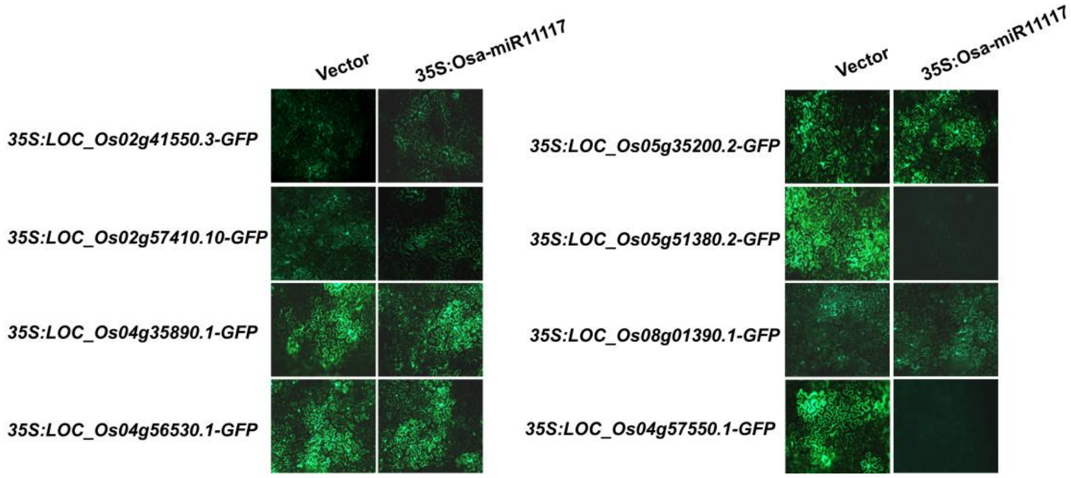

Fig. S2 Examination of target candidate genes of Osa-miR11117using GFP reporter assay in *N. benthamiana* leaves. The predicted target sites of the eight candidate genes were fused with green fluorescent protein (GFP), respectively, and the resultant constructs were coexpressed with 35S: Osa-miR11117 to *N. benthamiana* leaves using *Agrobacterium*-mediated infiltration. Images were then observed.

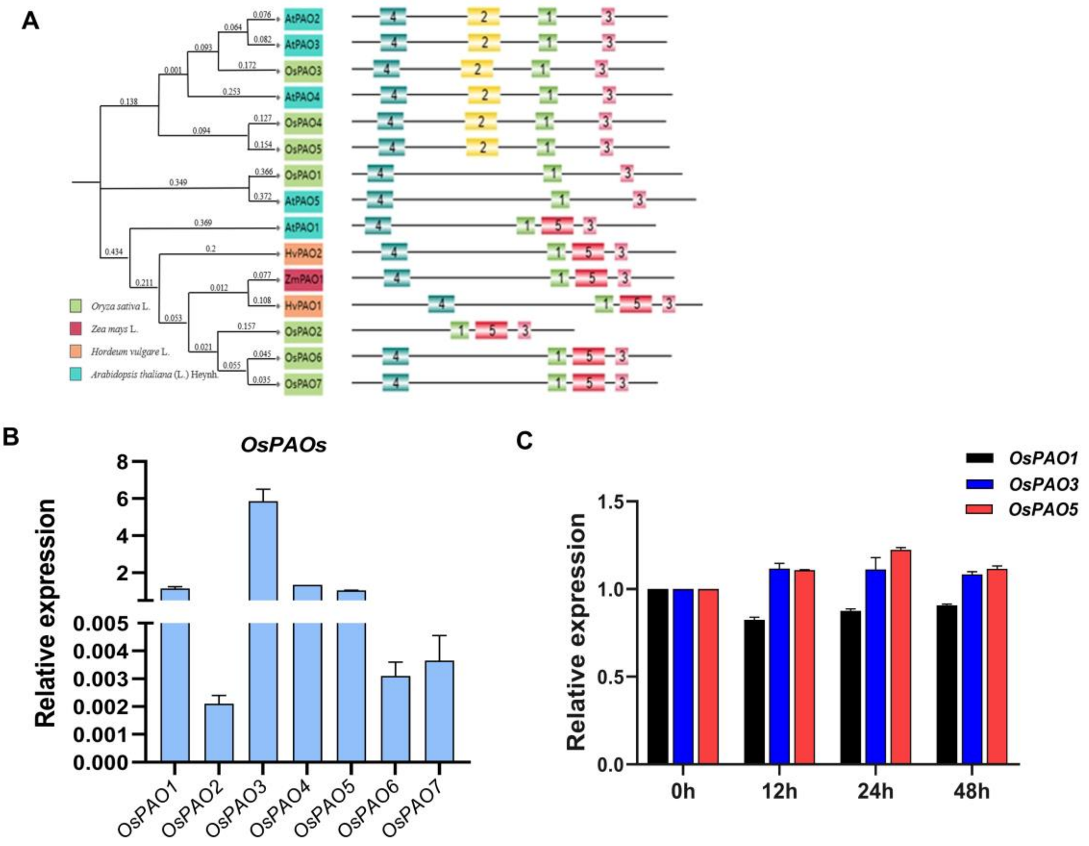

Fig. S3 Analysis of the evolutionary trees of *PAOs* in several different plants and the expression of *PAO* genes in rice leaves. (A) Phylogenetic analysis of polyamine oxidase genes in different plants. (B) The expression of the seven polyamine oxidase genes in rice leaves. (C) The expression of the three polyamine oxidase genes (*OsPAO1*, *OsPAO3* and *OsPAO5*) in rice leaves in response to infection by *M. oryzae*. Values are means of three replications. Error bars indicate SD.

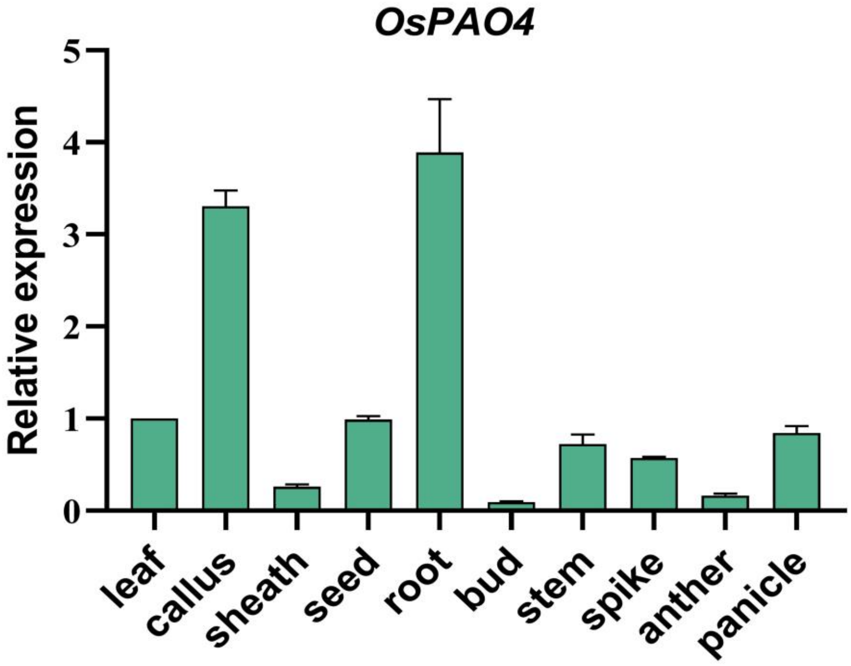

Fig. S4 Analysis of the tissue expression pattern of *OsPAO4*. qRT-PCR assay for the expression of *OsPAO4* in different tissues (leaf, callus, sheath, seed, root, bud, stem, spike, anther, and panicle).
