# Supplementary material for: Osa-miR11117 Targets OsPAO4 to Regulate Rice Immunity against the Blast Fungus Magnaporthe oryzae
Source: Int J Mol Sci. 2023 Nov 7;24(22):16052. doi: 10.3390/ijms242216052 (PMC10670930; doi:10.3390/ijms242216052)
Supplement: Supplementary file 1 [file ijms-24-16052-s001.zip › ijms-2683114-supplementary.pdf]

Table S1: List of Osa-miR11117 target genes

miRNA_Acc.	Target_Acc.	Expectation	UPE$	miRNA_start	miRNA_end	Target_start	Target_end	miRNA_aligned_fragment	alignment	Target_aligned_fragment	Inhibition	Target_Desc.	Multiplicity
GGAGGGAGAGGAAGAAGATGGGC	LOC_Os01g01860.1	3	-1	1	23	165	187	GGAGGGAGAGGAAGAAGAUGGGC	:.::::::::: ::::: ::.::	GUCCAUCUUCUUCCUCCCCUUCG	Cleavage	cDNA|transposon protein, putative, unclassified	1
GGAGGGAGAGGAAGAAGATGGGC	LOC_Os01g02660.1	3	-1	1	23	165	187	GGAGGGAGAGGAAGAAGAUGGGC	:.::::::::: ::::: ::.::	GUCCAUCUUCUUCCUCCCCUUCG	Cleavage	cDNA|retrotransposon protein, putative, unclassified	1
GGAGGGAGAGGAAGAAGATGGGC	LOC_Os01g04780.1	3	-1	1	23	165	187	GGAGGGAGAGGAAGAAGAUGGGC	:.::::::::: ::::: ::.::	GUCCAUCUUCUUCCUCCCCUUCG	Cleavage	cDNA|retrotransposon protein, putative, unclassified, expressed	1
GGAGGGAGAGGAAGAAGATGGGC	LOC_Os01g06110.1	3	-1	1	23	165	187	GGAGGGAGAGGAAGAAGAUGGGC	:.::::::::: ::::: ::.::	GUCCAUCUUCUUCCUCCCCUUCG	Cleavage	cDNA|transposon protein, putative, unclassified, expressed	1
GGAGGGAGAGGAAGAAGATGGGC	LOC_Os01g09160.1	3	-1	1	23	165	187	GGAGGGAGAGGAAGAAGAUGGGC	:.::::::::: ::::: ::.::	GUCCAUCUUCUUCCUCCCCUUCG	Cleavage	cDNA|transposon protein, putative, unclassified, expressed	1
GGAGGGAGA	LOC_Os	3	-1	1	23	165	187	GGAGGGAGAG	:.:::::::::	GUCCAUCUUC	Clea	cDNA|transposon	1

GGAAGAAGATGGGC	01g09530.1							GAAGAAGAUGGGC	::::: ::.::	UUCCUCCCCUUCG	vage	protein, putative, unclassified, expressed	
GGAGGGAGAGGAAGAAGATGGGC	LOC_Os01g10800.1	2	-1	1	23	28	50	GGAGGGAGAGGAAGAAGAUGGGC	::: :::::<br>:::.:::.:::<br>::	CCCCUUCUUCUUCUUCUUCCUCC	Cleavage	cDNA|pentatricopeptide, putative, expressed	1
GGAGGGAGAGGAAGAAGATGGGC	LOC_Os01g17370.1	2.5	-1	1	23	165	187	GGAGGGAGAGGAAGAAGAUGGGC	:.::.:::::<br>:::::::..::	GUCCGUCUUCUUCCUCUCUUUCG	Cleavage	cDNA|retrotransposon protein, putative, unclassified, expressed	1
GGAGGGAGAGGAAGAAGATGGGC	LOC_Os01g19200.1	2.5	-1	1	23	165	187	GGAGGGAGAGGAAGAAGAUGGGC	:.::.:::::<br>:::::::..::	GUCCGUCUUCUUCCUCUCUUUCG	Cleavage	cDNA|retrotransposon protein, putative, unclassified, expressed	1
GGAGGGAGAGGAAGAAGATGGGC	LOC_Os01g21820.1	0.5	-1	1	23	144	166	GGAGGGAGAGGAAGAAGAUGGGC	::::::::<br>:::::::.:::<br>::	ACCCAUCUUCUUCCUCUUCCUCC	Cleavage	cDNA|preprotein translocase subunit secA, chloroplast precursor, putative, expressed	1
GGAGGGAGAGGAAGAAGATGGGC	LOC_Os01g22410.1	3	-1	1	23	165	187	GGAGGGAGAGGAAGAAGAUGGGC	:.::::::::<br>::::: ::.::	GUCCAUCUUCUUCCUCCCCUUCG	Cleavage	cDNA|transposon protein, putative, unclassified, expressed	1
GGAGGGAGA	LOC_Os	3	-1	1	23	165	187	GGAGGGAGAG	:.::::::::	GUCCAUCUUC	Clea	cDNA|transposon	1

GGAAGAAGAT<br>GGGC	01g2274<br>0.1							GAAGAAGAUG<br>GGC	::::: ::.::	UUCCUCCCCU<br>UCG	vage	protein, putative,<br>unclassified,<br>expressed	
GGAGGGAGA<br>GGAAGAAGAT<br>GGGC	LOC_Os<br>01g2462<br>0.1	3	-1	1	23	165	187	GGAGGGAGAG<br>GAAGAAGAUG<br>GGC	:.::::::::<br>::::: ::.::	GUCCAUCUUC<br>UUCCUCCCCU<br>UCG	Clea<br>vage	cDNA|transposon<br>protein, putative,<br>unclassified	1
GGAGGGAGA<br>GGAAGAAGAT<br>GGGC	LOC_Os<br>01g2780<br>0.1	2.5	-1	1	23	165	187	GGAGGGAGAG<br>GAAGAAGAUG<br>GGC	:.::.:::::<br>:::::::..::	GUCCGUCUUC<br>UUCCUCUCUU<br>UCG	Clea<br>vage	cDNA|retrotranspo<br>son protein,<br>putative,<br>unclassified,<br>expressed	1
GGAGGGAGA<br>GGAAGAAGAT<br>GGGC	LOC_Os<br>01g2958<br>0.1	2.5	-1	1	23	165	187	GGAGGGAGAG<br>GAAGAAGAUG<br>GGC	:.::.:::::<br>:::::::..::	GUCCGUCUUC<br>UUCCUCUCUU<br>UCG	Clea<br>vage	cDNA|retrotranspo<br>son protein,<br>putative,<br>unclassified,<br>expressed	1
GGAGGGAGA<br>GGAAGAAGAT<br>GGGC	LOC_Os<br>01g3755<br>0.1	3	-1	1	23	165	187	GGAGGGAGAG<br>GAAGAAGAUG<br>GGC	:.::::::::<br>::::: ::.::	GUCCAUCUUC<br>UUCCUCCCCU<br>UCG	Clea<br>vage	cDNA|transposon<br>protein, putative,<br>unclassified,<br>expressed	1
GGAGGGAGA<br>GGAAGAAGAT<br>GGGC	LOC_Os<br>01g3788<br>0.1	3	-1	1	23	165	187	GGAGGGAGAG<br>GAAGAAGAUG<br>GGC	:.::::::::<br>::::: ::.::	GUCCAUCUUC<br>UUCCUCCCCU<br>UCG	Clea<br>vage	cDNA|transposon<br>protein, putative,<br>unclassified,<br>expressed	1
GGAGGGAGA<br>GGAAGAAGAT	LOC_Os<br>01g3793	2.5	-1	1	23	165	187	GGAGGGAGAG<br>GAAGAAGAUG	:.::::::::<br>::::: :::::	GUCCAUCUUC<br>UUCCUCCCCC	Clea<br>vage	cDNA|retrotranspo<br>son protein,	1

GGGC	0.1							GGC		UCG		putative, unclassified, expressed	
GGAGGGAGA GGAAGAAGAT GGGC	LOC_Os 01g3855 0.1	3	-1	1	23	165	187	GGAGGGAGAG GAAGAAGAUG GGC	:.:::::::: ::::: ::.::	GUCCAUCUUC UUCCUCCCCU UCG	Clea vage	cDNA|transposon protein, putative, unclassified, expressed	1
GGAGGGAGA GGAAGAAGAT GGGC	LOC_Os 01g4106 0.1	2.5	-1	1	23	165	187	GGAGGGAGAG GAAGAAGAUG GGC	:.::.::::: :::::::..::	GUCCGUCUUC UUCCUCUCUU UCG	Clea vage	cDNA|retrotranspo son protein, putative, unclassified, expressed	1
GGAGGGAGA GGAAGAAGAT GGGC	LOC_Os 01g4558 0.1	3	-1	1	23	165	187	GGAGGGAGAG GAAGAAGAUG GGC	:.:::::::: ::::: ::.::	GUCCAUCUUC UUCCUCCCCU UCG	Clea vage	cDNA|transposon protein, putative, unclassified	1
GGAGGGAGA GGAAGAAGAT GGGC	LOC_Os 01g4678 0.1	3	-1	1	23	165	187	GGAGGGAGAG GAAGAAGAUG GGC	:.:::::::: ::::: ::.::	GUCCAUCUUC UUCCUCCCCU UCG	Clea vage	cDNA|transposon protein, putative, unclassified, expressed	1
GGAGGGAGA GGAAGAAGAT GGGC	LOC_Os 01g4702 0.1	3	-1	1	23	165	187	GGAGGGAGAG GAAGAAGAUG GGC	:.:::::::: ::::: ::.::	GUCCAUCUUC UUCCUCCCCU UCG	Clea vage	cDNA|transposon protein, putative, unclassified, expressed	1
GGAGGGAGA GGAAGAAGAT GGGC	LOC_Os 01g4997 0.1	2.5	-1	1	23	165	187	GGAGGGAGAG GAAGAAGAUG GGC	:.::.::::: :::::::..::	GUCCGUCUUC UUCCUCUCUU UCG	Clea vage	cDNA|retrotranspo son protein, putative,	1

												unclassified, expressed	
GGAGGGAGAGGAAGAAGATGGGC	LOC_Os01g50650.1	2.5	-1	1	23	165	187	GGAGGGAGAGGAAGAAGAUGGGC	:.::.:::::: :::::::..::	GUCCGUCUUCUUCCUCUCUUUCG	Cleavage	cDNA|retrotransposon protein, putative, unclassified, expressed	1
GGAGGGAGAGGAAGAAGATGGGC	LOC_Os01g54680.1	2.5	-1	1	23	165	187	GGAGGGAGAGGAAGAAGAUGGGC	:.::.:::::: :::::::..::	GUCCGUCUUCUUCCUCUCUUUCG	Cleavage	cDNA|retrotransposon protein, putative, unclassified, expressed	1
GGAGGGAGAGGAAGAAGATGGGC	LOC_Os01g61000.1	3	-1	1	23	165	187	GGAGGGAGAGGAAGAAGAUGGGC	:.::::::::: ::::: ::.::	GUCCAUCUUCUUCCUCCCCUUCG	Cleavage	cDNA|transposon protein, putative, unclassified, expressed	1
GGAGGGAGAGGAAGAAGATGGGC	LOC_Os01g63140.1	2.5	-1	1	23	165	187	GGAGGGAGAGGAAGAAGAUGGGC	:.::.:::::: :::::::..::	GUCCGUCUUCUUCCUCUCUUUCG	Cleavage	cDNA|retrotransposon protein, putative, unclassified	1
GGAGGGAGAGGAAGAAGATGGGC	LOC_Os01g71510.1	3	-1	1	23	165	187	GGAGGGAGAGGAAGAAGAUGGGC	:.::::::::: ::::: ::.::	GUCCAUCUUCUUCCUCCCCUUCG	Cleavage	cDNA|transposon protein, putative, unclassified, expressed	1
GGAGGGAGAGGAAGAAGAT	LOC_Os01g7405	3	-1	1	23	165	187	GGAGGGAGAGGAAGAAGAUG	:.::::::::: ::::: ::.::	GUCCAUCUUCUUCCUCCCCU	Cleavage	cDNA|transposon protein, putative,	1

GGGC	0.1							GGC		UCG		unclassified	
GGAGGGAGA<br>GGAAGAAGAT<br>GGGC	LOC_Os<br>02g0414<br>0.1	3	-1	1	23	165	187	GGAGGGAGAG<br>GAAGAAGAUG<br>GGC	:.:::::::::<br>::::: ::.::	GUCCAUCUUC<br>UUCCUCCCCU<br>UCG	Clea<br>vage	cDNA|retrotranspo<br>son protein,<br>putative,<br>unclassified,<br>expressed	1
GGAGGGAGA<br>GGAAGAAGAT<br>GGGC	LOC_Os<br>02g0429<br>0.1	2.5	-1	1	23	165	187	GGAGGGAGAG<br>GAAGAAGAUG<br>GGC	:.::.::::::<br>:::::::..::	GUCCGUCUUC<br>UUCCUCUCUU<br>UCG	Clea<br>vage	cDNA|retrotranspo<br>son protein,<br>putative,<br>unclassified,<br>expressed	1
GGAGGGAGA<br>GGAAGAAGAT<br>GGGC	LOC_Os<br>02g0604<br>0.1	3	-1	1	23	165	187	GGAGGGAGAG<br>GAAGAAGAUG<br>GGC	:.:::::::::<br>::::: ::.::	GUCCAUCUUC<br>UUCCUCCCCU<br>UCG	Clea<br>vage	cDNA|retrotranspo<br>son protein,<br>putative,<br>unclassified,<br>expressed	1
GGAGGGAGA<br>GGAAGAAGAT<br>GGGC	LOC_Os<br>02g1742<br>0.1	2.5	-1	1	23	165	187	GGAGGGAGAG<br>GAAGAAGAUG<br>GGC	:.::.::::::<br>:::::::..::	GUCCGUCUUC<br>UUCCUCUCUU<br>UCG	Clea<br>vage	cDNA|retrotranspo<br>son protein,<br>putative,<br>unclassified,<br>expressed	1
GGAGGGAGA<br>GGAAGAAGAT<br>GGGC	LOC_Os<br>02g2242<br>0.1	3	-1	1	23	165	187	GGAGGGAGAG<br>GAAGAAGAUG<br>GGC	:.::.::::::<br>:::::::..:.	GUCCGUCUUC<br>UUCCUCUCUU<br>UUG	Clea<br>vage	cDNA|retrotranspo<br>son protein,<br>putative,<br>unclassified,<br>expressed	1

GGAGGGAGA GGAAGAAGAT GGGC	LOC_Os 02g3417 0.1	3	-1	1	23	165	187	GGAGGGAGAG GAAGAAGAUG GGC	:.:::::::: ::::: ::.::	GUCCAUCUUC UUCCUCCCCU UCG	Clea vage	cDNA|transposon protein, putative, unclassified, expressed	1
GGAGGGAGA GGAAGAAGAT GGGC	LOC_Os 02g3670 0.1	1.5	-1	1	23	32	54	GGAGGGAGAG GAAGAAGAUG GGC	::.:.::::: ::::::.:::. :	GCUCGUCUUC UUCCUCUUCC UUC	Clea vage	cDNA|sucrose transporter BoSUT1, putative, expressed	1
GGAGGGAGA GGAAGAAGAT GGGC	LOC_Os 02g3930 0.1	1.5	-1	1	23	1	23	GGAGGGAGAG GAAGAAGAUG GGC	::.:::: ::::.:::.:: :::	AGCCGUCUUC UUCUUCUUCC UCC	Clea vage	cDNA|RNA-binding protein Luc7-like, putative, expressed	1
GGAGGGAGA GGAAGAAGAT GGGC	LOC_Os 02g4155 0.3	1	-1	1	23	308	330	GGAGGGAGAG GAAGAAGAUG GGC	::.::::::: ::.:::.:::: :	GCUCAUCUUC UUCUUCUUCC UCC	Clea vage	cDNA|FAD binding domain of DNA photolyase domain containing protein, expressed	1
GGAGGGAGA GGAAGAAGAT GGGC	LOC_Os 02g4841 0.1	2.5	-1	1	23	165	187	GGAGGGAGAG GAAGAAGAUG GGC	:.::.:::::: ::::::...::	GUCCGUCUUC UUCCUCUCUU UCG	Clea vage	cDNA|retrotransposon protein, putative, unclassified, expressed	1
GGAGGGAGA GGAAGAAGAT GGGC	LOC_Os 02g5207 0.1	2	-1	1	23	165	187	GGAGGGAGAG GAAGAAGAUG GGC	:.::::::::: ::::::...::	GUCCAUCUUC UUCCUCUCUU UCG	Clea vage	cDNA|retrotransposon protein, putative, unclassified,	1

												expressed	
GGAGGGAGAGGAAGAAGATGGGC	LOC_Os02g57410.1	1	-1	1	23	65	87	GGAGGGAGAGGAAGAAGAUGGGC	:.::::::::::.:::::::::.	UCUCAUCUUCUUCUUCUCCCUCU	Cleavage	cDNA|OTU-like cysteine protease family protein, putative, expressed	1
GGAGGGAGAGGAAGAAGATGGGC	LOC_Os02g57410.10	1	-1	1	23	65	87	GGAGGGAGAGGAAGAAGAUGGGC	:.::::::::::.:::::::::.	UCUCAUCUUCUUCUUCUCCCUCU	Cleavage	cDNA|OTU-like cysteine protease family protein, putative, expressed	1
GGAGGGAGAGGAAGAAGATGGGC	LOC_Os02g57410.2	1	-1	1	23	65	87	GGAGGGAGAGGAAGAAGAUGGGC	:.::::::::::.:::::::::.	UCUCAUCUUCUUCUUCUCCCUCU	Cleavage	cDNA|OTU-like cysteine protease family protein, putative, expressed	1
GGAGGGAGAGGAAGAAGATGGGC	LOC_Os02g57410.3	1	-1	1	23	65	87	GGAGGGAGAGGAAGAAGAUGGGC	:.::::::::::.:::::::::.	UCUCAUCUUCUUCUUCUCCCUCU	Cleavage	cDNA|OTU-like cysteine protease family protein, putative, expressed	1
GGAGGGAGAGGAAGAAGATGGGC	LOC_Os02g57410.4	1	-1	1	23	65	87	GGAGGGAGAGGAAGAAGAUGGGC	:.::::::::::.:::::::::.	UCUCAUCUUCUUCUUCUCCCUCU	Cleavage	cDNA|OTU-like cysteine protease family protein, putative, expressed	1
GGAGGGAGAGGAAGAAGATGGGC	LOC_Os02g57410.5	1	-1	1	23	65	87	GGAGGGAGAGGAAGAAGAUGGGC	:.::::::::::.:::::::::.	UCUCAUCUUCUUCUUCUCCCUCU	Cleavage	cDNA|OTU-like cysteine protease family protein, putative, expressed	1
GGAGGGAGA	LOC_Os	1	-1	1	23	65	87	GGAGGGAGAG	:.:::::::	UCUCAUCUUC	Clea	cDNA|OTU-like	1

GGAAGAAGAT GGGC	02g5741 0.6							GAAGAAGAUG GGC	:::.:::::::: :.	UUCUUCUCCC UCU	vage	cysteine protease family protein, putative, expressed	
GGAGGGAGA GGAAGAAGAT GGGC	LOC_Os 02g5741 0.7	1	-1	1	23	65	87	GGAGGGAGAG GAAGAAGAUG GGC	:.:::::: :::.:::::::: :.	UCUCAUCUUC UUCUUCUCCC UCU	Clea vage	cDNA|OTU-like cysteine protease family protein, putative, expressed	1
GGAGGGAGA GGAAGAAGAT GGGC	LOC_Os 02g5741 0.8	1	-1	1	23	65	87	GGAGGGAGAG GAAGAAGAUG GGC	:.:::::: :::.:::::::: :.	UCUCAUCUUC UUCUUCUCCC UCU	Clea vage	cDNA|OTU-like cysteine protease family protein, putative, expressed	1
GGAGGGAGA GGAAGAAGAT GGGC	LOC_Os 02g5741 0.9	1	-1	1	23	65	87	GGAGGGAGAG GAAGAAGAUG GGC	:.:::::: :::.:::::::: :.	UCUCAUCUUC UUCUUCUCCC UCU	Clea vage	cDNA|OTU-like cysteine protease family protein, putative, expressed	1
GGAGGGAGA GGAAGAAGAT GGGC	LOC_Os 03g0106 0.1	2.5	-1	1	23	165	187	GGAGGGAGAG GAAGAAGAUG GGC	:.::.:::::: :::::::..::	GUCCGUCUUC UUCCUCUCUU UCG	Clea vage	cDNA|retrotranspo son protein, putative, unclassified, expressed	1
GGAGGGAGA GGAAGAAGAT GGGC	LOC_Os 03g0140 0.1	2.5	-1	1	23	165	187	GGAGGGAGAG GAAGAAGAUG GGC	:.::.:::::: :::::::..::	GUCCGUCUUC UUCCUCUCUU UCG	Clea vage	cDNA|retrotranspo son protein, putative, unclassified, expressed	1
GGAGGGAGA	LOC_Os	2.5	-1	1	23	165	187	GGAGGGAGAG	:.::.::::::	GUCCGUCUUC	Clea	cDNA|retrotranspo	1

GGAAGAAGAT<br>GGGC	03g0500<br>0.1							GAAGAAGAUG<br>GGC	:::::::..::	UUCCUCUCUU<br>UCG	vage	son protein,<br>putative,<br>unclassified,<br>expressed	
GGAGGGAGA<br>GGAAGAAGAT<br>GGGC	LOC_Os<br>03g0627<br>0.1	3	-1	1	23	165	187	GGAGGGAGAG<br>GAAGAAGAUG<br>GGC	:.::.::::::<br>::::.:::.:.	GUCCGUCUUC<br>UUCCUUUCCU<br>UUG	Clea<br>vage	cDNA|retrotranspo<br>son protein,<br>putative,<br>unclassified,<br>expressed	1
GGAGGGAGA<br>GGAAGAAGAT<br>GGGC	LOC_Os<br>03g0702<br>0.1	3	-1	1	23	165	187	GGAGGGAGAG<br>GAAGAAGAUG<br>GGC	:.:::::::::<br>::::: ::.::	GUCCAUCUUC<br>UUCCUCCCCU<br>UCG	Clea<br>vage	cDNA|retrotranspo<br>son protein,<br>putative,<br>unclassified,<br>expressed	1
GGAGGGAGA<br>GGAAGAAGAT<br>GGGC	LOC_Os<br>03g1561<br>0.1	2.5	-1	1	23	165	187	GGAGGGAGAG<br>GAAGAAGAUG<br>GGC	:.::.::::::<br>:::::::..::	GUCCGUCUUC<br>UUCCUCUCUU<br>UCG	Clea<br>vage	cDNA|retrotranspo<br>son protein,<br>putative,<br>unclassified,<br>expressed	1
GGAGGGAGA<br>GGAAGAAGAT<br>GGGC	LOC_Os<br>03g2428<br>0.1	3	-1	1	23	165	187	GGAGGGAGAG<br>GAAGAAGAUG<br>GGC	:.:::::::::<br>::::: ::.::	GUCCAUCUUC<br>UUCCUCCCCU<br>UCG	Clea<br>vage	cDNA|transposon<br>protein, putative,<br>unclassified,<br>expressed	1
GGAGGGAGA<br>GGAAGAAGAT<br>GGGC	LOC_Os<br>03g2437<br>0.1	3	-1	1	23	165	187	GGAGGGAGAG<br>GAAGAAGAUG<br>GGC	:.:::::::::<br>::::: ::.::	GUCCAUCUUC<br>UUCCUCCCCU<br>UCG	Clea<br>vage	cDNA|transposon<br>protein, putative,<br>unclassified,	1

												expressed	
GGAGGGAGA GGAAGAAGAT GGGC	LOC_Os 03g3667 0.1	2.5	-1	1	23	165	187	GGAGGGAGAG GAAGAAGAUG GGC	:.::.:::::: ::::::..::	GUCCGUCUUC UUCCUCUCUU UCG	Clea vage	cDNA|retrotranspo son protein, putative, Ty3-gypsy subclass, expressed	1
GGAGGGAGA GGAAGAAGAT GGGC	LOC_Os 03g4199 0.1	2.5	-1	1	23	165	187	GGAGGGAGAG GAAGAAGAUG GGC	:.::.:::::: ::::::..::	GUCCGUCUUC UUCCUCUCUU UCG	Clea vage	cDNA|retrotranspo son protein, putative, unclassified, expressed	1
GGAGGGAGA GGAAGAAGAT GGGC	LOC_Os 03g4440 0.1	2	-1	1	23	165	187	GGAGGGAGAG GAAGAAGAUG GGC	:.::.:::::: :::::::.::	GUCCGUCUUC UUCCUCUCCU UCG	Clea vage	cDNA|retrotranspo son protein, putative, unclassified, expressed	1
GGAGGGAGA GGAAGAAGAT GGGC	LOC_Os 03g4759 0.1	2.5	-1	1	23	165	187	GGAGGGAGAG GAAGAAGAUG GGC	:.::.:::::: ::::::..::	GUCCGUCUUC UUCCUCUCUU UCG	Clea vage	cDNA|retrotranspo son protein, putative, Ty3-gypsy subclass, expressed	1
GGAGGGAGA GGAAGAAGAT GGGC	LOC_Os 03g5944 0.1	1	-1	1	23	66	88	GGAGGGAGAG GAAGAAGAUG GGC	.:: :::: :::::::::: ::	AUCCUUCUUC UUCCUCUCCC UCC	Clea vage	cDNA|dirigent, putative, expressed	1
GGAGGGAGA GGAAGAAGAT	LOC_Os 03g6364	3	-1	1	23	165	187	GGAGGGAGAG GAAGAAGAUG	:.:::::::: :::: ::.::	GUCCAUCUUC UUCCUCCCCU	Clea vage	cDNA|transposon protein, putative,	1

GGGC	0.1							GGC		UCG		unclassified, expressed	
GGAGGGAGA GGAAGAAGAT GGGC	LOC_Os 03g6380 0.1	3	-1	1	23	165	187	GGAGGGAGAG GAAGAAGAUG GGC	:.::::::::: ::::: ::.::	GUCCAUCUUC UUCCUCCCCU UCG	Clea vage	cDNA|transposon protein, putative, unclassified, expressed	1
GGAGGGAGA GGAAGAAGAT GGGC	LOC_Os 04g0553 0.1	3	-1	1	23	165	187	GGAGGGAGAG GAAGAAGAUG GGC	:.::::::::: ::::: ::.::	GUCCAUCUUC UUCCUCCCCU UCG	Clea vage	cDNA|transposon protein, putative, unclassified, expressed	1
GGAGGGAGA GGAAGAAGAT GGGC	LOC_Os 04g1117 0.1	2.5	-1	1	23	165	187	GGAGGGAGAG GAAGAAGAUG GGC	:.::.:::::: :::::::..::	GUCCGUCUUC UUCCUCUCUU UCG	Clea vage	cDNA|retrotranspo son protein, putative, unclassified, expressed	1
GGAGGGAGA GGAAGAAGAT GGGC	LOC_Os 04g1212 0.1	1.5	-1	1	23	294	316	GGAGGGAGAG GAAGAAGAUG GGC	:..:::::::: :::::::..:: .	GUUCAUCUUC UUCCUCUCUU UCU	Clea vage	cDNA|transposon protein, putative, unclassified, expressed	1
GGAGGGAGA GGAAGAAGAT GGGC	LOC_Os 04g1278 0.1	2.5	-1	1	23	165	187	GGAGGGAGAG GAAGAAGAUG GGC	:.::.:::::: :::::::..::	GUCCGUCUUC UUCCUCUCUU UCG	Clea vage	cDNA|retrotranspo son protein, putative, unclassified	1
GGAGGGAGA GGAAGAAGAT GGGC	LOC_Os 04g1727 0.1	3	-1	1	23	165	187	GGAGGGAGAG GAAGAAGAUG GGC	:.::::::::: ::::: ::.::	GUCCAUCUUC UUCCUCCCCU UCG	Clea vage	cDNA|transposon protein, putative, unclassified,	1

												expressed	
GGAGGGAGAGGAAGAAGATGGGC	LOC_Os04g20054.1	2.5	-1	1	23	165	187	GGAGGGAGAGGAAGAAGAUGGGC	:.::.::::::::::::..::	GUCCGUCUUCUUCCUCUCUUUCG	Cleavage	cDNA|retrotransposon protein, putative, unclassified, expressed	1
GGAGGGAGAGGAAGAAGATGGGC	LOC_Os04g21230.1	3	-1	1	23	165	187	GGAGGGAGAGGAAGAAGAUGGGC	:.:::::::::::: ::.::	GUCCAUCUUCUUCCUCCCCUUCG	Cleavage	cDNA|retrotransposon protein, putative, unclassified, expressed	1
GGAGGGAGAGGAAGAAGATGGGC	LOC_Os04g21260.1	3	-1	1	23	165	187	GGAGGGAGAGGAAGAAGAUGGGC	:.:::::::::::: ::.::	GUCCAUCUUCUUCCUCCCCUUCG	Cleavage	cDNA|transposon protein, putative, unclassified, expressed	1
GGAGGGAGAGGAAGAAGATGGGC	LOC_Os04g23880.1	3	-1	1	23	165	187	GGAGGGAGAGGAAGAAGAUGGGC	:.:::::::::::: ::.::	GUCCAUCUUCUUCCUCCCCUUCG	Cleavage	cDNA|transposon protein, putative, unclassified, expressed	1
GGAGGGAGAGGAAGAAGATGGGC	LOC_Os04g27220.1	3	-1	1	23	165	187	GGAGGGAGAGGAAGAAGAUGGGC	:.:::::::::::: ::.::	GUCCAUCUUCUUCCUCCCCUUCG	Cleavage	cDNA|transposon protein, putative, unclassified, expressed	1
GGAGGGAGAGGAAGAAGATGGGC	LOC_Os04g27870.1	2.5	-1	1	23	165	187	GGAGGGAGAGGAAGAAGAUGGGC	:.::.::::::::::::..::	GUCCGUCUUCUUCCUCUCUUUCG	Cleavage	cDNA|retrotransposon protein, putative,	1

												unclassified	
GGAGGGAGAGGAAGAAGATGGGC	LOC_Os04g31850.1	3	-1	1	23	165	187	GGAGGGAGAGGAAGAAGAUGGGC	:.::.:::::: ::::.::..::	GUCCGUCUUCUUCCUUUCUUUCG	Cleavage	cDNA|retrotransposon protein, putative, unclassified, expressed	1
GGAGGGAGAGGAAGAAGATGGGC	LOC_Os04g35890.1	2	-1	1	23	11	33	GGAGGGAGAGGAAGAAGAUGGGC	.:::::::: :::::: .::: ::	CUCCAUCUUCUUCCUCCUCCUCC	Cleavage	cDNA|TKL_IRAK_CR4L.3 - The CR4L subfamily has homology with Crinkly4, expressed	1
GGAGGGAGAGGAAGAAGATGGGC	LOC_Os04g38070.1	2.5	-1	1	23	165	187	GGAGGGAGAGGAAGAAGAUGGGC	:.::.:::::: :::::::..::	GUCCGUCUUCUUCCUCUCUUUCG	Cleavage	cDNA|retrotransposon protein, putative, unclassified, expressed	1
GGAGGGAGAGGAAGAAGATGGGC	LOC_Os04g43930.1	2.5	-1	1	23	165	187	GGAGGGAGAGGAAGAAGAUGGGC	:.::.:::::: :::::::..::	GUCCGUCUUCUUCCUCUCUUUCG	Cleavage	cDNA|retrotransposon protein, putative, unclassified	1
GGAGGGAGAGGAAGAAGATGGGC	LOC_Os04g48080.1	2.5	-1	1	23	165	187	GGAGGGAGAGGAAGAAGAUGGGC	:.::.:::::: :::::::..::	GUCCGUCUUCUUCCUCUCUUUCG	Cleavage	cDNA|retrotransposon protein, putative, unclassified, expressed	1

GGAGGGAGAGGAAGAAGATGGGC	LOC_Os04g48390.1	1.5	-1	1	23	29	51	GGAGGGAGAGGAAGAAGAUGGGC	::::.:::::: ::.:::.:::: :	GCCCGUCUUCUUCUUCUUCCUCC	Cleavage	cDNA|uncharacterized membrane protein, putative, expressed	1
GGAGGGAGAGGAAGAAGATGGGC	LOC_Os04g51200.1	3	-1	1	23	165	187	GGAGGGAGAGGAAGAAGAUGGGC	:.::::::::: ::::: ::.::	GUCCAUCUUCUUCCUCCCCUUCG	Cleavage	cDNA|retrotransposon protein, putative, unclassified, expressed	1
GGAGGGAGAGGAAGAAGATGGGC	LOC_Os04g54270.1	3	-1	1	23	165	187	GGAGGGAGAGGAAGAAGAUGGGC	:.::::::::: ::::: ::.::	GUCCAUCUUCUUCCUCCCCUUCG	Cleavage	cDNA|transposon protein, putative, unclassified, expressed	1
GGAGGGAGAGGAAGAAGATGGGC	LOC_Os04g54690.1	3	-1	1	23	165	187	GGAGGGAGAGGAAGAAGAUGGGC	:.::::::::: ::::: ::.::	GUCCAUCUUCUUCCUCCCCUUCG	Cleavage	cDNA|retrotransposon protein, putative, unclassified, expressed	1
GGAGGGAGAGGAAGAAGATGGGC	LOC_Os04g55870.1	3	-1	1	23	165	187	GGAGGGAGAGGAAGAAGAUGGGC	:.::::::::: ::::: ::.::	GUCCAUCUUCUUCCUCCCCUUCG	Cleavage	cDNA|transposon protein, putative, unclassified, expressed	1
GGAGGGAGAGGAAGAAGATGGGC	LOC_Os04g56530.1	1.5	-1	1	23	73	95	GGAGGGAGAGGAAGAAGAUGGGC	.::.::::: :::.:::.::: ::	CUCCGUCUUCUUCUUCUUCCUCC	Cleavage	cDNA|STE_MEKK_ste11_MAP3K.1 - STE kinases include homologs	1

												to sterile 7, sterile 11 and sterile 20 from yeast, expressed	
GGAGGGAGAGGAAGAAGATGGGC	LOC_Os04g57550.1	1.5	-1	1	23	394	416	GGAGGGAGAGGAAGAAGAUGGGC	:..:::::::::.:::.:.:::	GUUCAUCUUCUUCUUCUUCUUCC	Cleavage	cDNA|amine oxidase, flavin-containing, domain containing protein, expressed	1
GGAGGGAGAGGAAGAAGATGGGC	LOC_Os05g04030.1	2.5	-1	1	23	165	187	GGAGGGAGAGGAAGAAGAUGGGC	:.::.:::::::::.:::.::	GUCCGUCUUCUUCCUUUCCUUCG	Cleavage	cDNA|retrotransposon protein, putative, unclassified, expressed	1
GGAGGGAGAGGAAGAAGATGGGC	LOC_Os05g04290.1	2.5	-1	1	23	165	187	GGAGGGAGAGGAAGAAGAUGGGC	:.::.::::::::::::..::	GUCCGUCUUCUUCCUCUCUUUCG	Cleavage	cDNA|retrotransposon protein, putative, unclassified, expressed	1
GGAGGGAGAGGAAGAAGATGGGC	LOC_Os05g06860.1	3	-1	1	23	165	187	GGAGGGAGAGGAAGAAGAUGGGC	:.:::::::::::: ::.::	GUCCAUCUUCUUCCUCCCCUUCG	Cleavage	cDNA|transposon protein, putative, unclassified, expressed	1
GGAGGGAGAGGAAGAAGATGGGC	LOC_Os05g10260.1	2.5	-1	1	23	165	187	GGAGGGAGAGGAAGAAGAUGGGC	:.::.::::::::::::..::	GUCCGUCUUCUUCCUCUCUUUCG	Cleavage	cDNA|retrotransposon protein, putative,	1

												unclassified, expressed	
GGAGGGAGAGGAAGAAGATGGGC	LOC_Os05g14350.1	3	-1	1	23	165	187	GGAGGGAGAGGAAGAAGAUGGGC	:.::::::::::::: ::.::	GUCCAUCUUCUUCCUCCCCUUCG	Cleavage	cDNA|retrotransposon protein, putative, unclassified, expressed	1
GGAGGGAGAGGAAGAAGATGGGC	LOC_Os05g16130.1	3	-1	1	23	165	187	GGAGGGAGAGGAAGAAGAUGGGC	:.::::::::::::: ::.::	GUCCAUCUUCUUCCUCCCCUUCG	Cleavage	cDNA|transposon protein, putative, unclassified, expressed	1
GGAGGGAGAGGAAGAAGATGGGC	LOC_Os05g19160.1	3	-1	1	23	165	187	GGAGGGAGAGGAAGAAGAUGGGC	:.::::::::::::: ::.::	GUCCAUCUUCUUCCUCCCCUUCG	Cleavage	cDNA|transposon protein, putative, unclassified, expressed	1
GGAGGGAGAGGAAGAAGATGGGC	LOC_Os05g20390.1	3	-1	1	23	165	187	GGAGGGAGAGGAAGAAGAUGGGC	:.::::::::::::: ::.::	GUCCAUCUUCUUCCUCCCCUUCG	Cleavage	cDNA|transposon protein, putative, unclassified, expressed	1
GGAGGGAGAGGAAGAAGATGGGC	LOC_Os05g21110.1	2.5	-1	1	23	165	187	GGAGGGAGAGGAAGAAGAUGGGC	:.::.:::::::::::..::	GUCCGUCUUCUUCCUCUCUUUCG	Cleavage	cDNA|retrotransposon protein, putative, unclassified, expressed	1
GGAGGGAGAGGAAGAAGAT	LOC_Os05g2336	3	-1	1	23	165	187	GGAGGGAGAGGAAGAAGAUG	:.::::::::::::: ::.::	GUCCAUCUUCUUCCUCCCCU	Cleavage	cDNA|transposon protein, putative,	1

GGGC	0.1							GGC		UCG		unclassified	
GGAGGGAGA GGAAGAAGAT GGGC	LOC_Os 05g2659 0.1	2.5	-1	1	23	165	187	GGAGGGAGAG GAAGAAGAUG GGC	:.::.::::: :::::::..::	GUCCGUCUUC UUCCUCUCUU UCG	Clea vage	cDNA|retrotranspo son protein, putative, unclassified, expressed	1
GGAGGGAGA GGAAGAAGAT GGGC	LOC_Os 05g2891 0.1	2.5	-1	1	23	165	187	GGAGGGAGAG GAAGAAGAUG GGC	:.::.::::: :::::::..::	GUCCGUCUUC UUCCUCUCUU UCG	Clea vage	cDNA|retrotranspo son protein, putative, unclassified, expressed	1
GGAGGGAGA GGAAGAAGAT GGGC	LOC_Os 05g3055 0.1	2.5	-1	1	23	165	187	GGAGGGAGAG GAAGAAGAUG GGC	:.::.::::: :::::::..::	GUCCGUCUUC UUCCUCUCUU UCG	Clea vage	cDNA|retrotranspo son protein, putative, unclassified, expressed	1
GGAGGGAGA GGAAGAAGAT GGGC	LOC_Os 05g3300 0.2	2	-1	1	23	115	137	GGAGGGAGAG GAAGAAGAUG GGC	:.:::::: :::.:::.::: :	CCUCAUCUUC UUCUUCUUCC UCG	Clea vage	cDNA|E3 ubiquitin-protein ligase MGRN1, putative, expressed	1
GGAGGGAGA GGAAGAAGAT GGGC	LOC_Os 05g3520 0.2	1	-1	1	23	237	259	GGAGGGAGAG GAAGAAGAUG GGC	.::::: ::::.:::.:: :::	CAUCAUCUUC UUCUUCUUCC UCC	Clea vage	cDNA|glycosyl transferase, putative, expressed	1
GGAGGGAGA GGAAGAAGAT GGGC	LOC_Os 05g3758 0.1	3	-1	1	23	165	187	GGAGGGAGAG GAAGAAGAUG GGC	:.::.::::: :::.::..::	GUCCGUCUUC UUCCUUUCUU UCG	Clea vage	cDNA|retrotranspo son protein, putative,	1

												unclassified, expressed	
GGAGGGAGAGGAAGAAGATGGGC	LOC_Os05g41690.1	2.5	-1	1	23	165	187	GGAGGGAGAGGAAGAAGAUGGGC	:.::.::::: :::::::..::	GUCCGUCUUCUUCCUCUCUUUCG	Cleavage	cDNA|retrotransposon protein, putative, unclassified, expressed	1
GGAGGGAGAGGAAGAAGATGGGC	LOC_Os05g42950.1	3	-1	1	23	165	187	GGAGGGAGAGGAAGAAGAUGGGC	:.:::::::: ::::: ::.::	GUCCAUCUUCUUCCUCCCCUUCG	Cleavage	cDNA|retrotransposon protein, putative, unclassified	1
GGAGGGAGAGGAAGAAGATGGGC	LOC_Os05g45790.1	3	-1	1	23	165	187	GGAGGGAGAGGAAGAAGAUGGGC	:.:::::::: ::::: ::.::	GUCCAUCUUCUUCCUCCCCUUCG	Cleavage	cDNA|retrotransposon protein, putative, unclassified, expressed	1
GGAGGGAGAGGAAGAAGATGGGC	LOC_Os05g51380.1	2.5	-1	1	23	560	582	GGAGGGAGAGGAAGAAGAUGGGC	:::::.::: :: ::::::: :.	CCCCAUUUUCUUGCUCUCCCUCU	Translation	cDNA|expressed protein	1
GGAGGGAGAGGAAGAAGATGGGC	LOC_Os05g51380.2	2.5	-1	1	23	420	442	GGAGGGAGAGGAAGAAGAUGGGC	:::::.::: :: ::::::: :.	CCCCAUUUUCUUGCUCUCCCUCU	Translation	cDNA|expressed protein	1
GGAGGGAGAGGAAGAAGATGGGC	LOC_Os06g15920.1	3	-1	1	23	165	187	GGAGGGAGAGGAAGAAGAUGGGC	:.:::::::: ::::: ::.::	GUCCAUCUUCUUCCUCCCCUUCG	Cleavage	cDNA|transposon protein, putative, unclassified, expressed	1

GGAGGGAGAGGAAGAAGATGGGC	LOC_Os06g16220.1	3	-1	1	23	165	187	GGAGGGAGAGGAAGAAGAUGGGC	:.::::::::: ::::: ::.::	GUCCAUCUUCUUCCUCCCCUUCG	Cleavage	cDNA|transposon protein, putative, unclassified, expressed	1
GGAGGGAGAGGAAGAAGATGGGC	LOC_Os06g17210.1	3	-1	1	23	165	187	GGAGGGAGAGGAAGAAGAUGGGC	:.::::::::: ::::: ::.::	GUCCAUCUUCUUCCUCCCCUUCG	Cleavage	cDNA|transposon protein, putative, unclassified, expressed	1
GGAGGGAGAGGAAGAAGATGGGC	LOC_Os06g20160.1	2	-1	1	23	165	187	GGAGGGAGAGGAAGAAGAUGGGC	:.::::::::: :::::::..::	GUCCAUCUUCUUCCUCUCUUUCG	Cleavage	cDNA|retrotransposon protein, putative, Ty3-gypsy subclass, expressed	1
GGAGGGAGAGGAAGAAGATGGGC	LOC_Os06g23820.1	2.5	-1	1	23	165	187	GGAGGGAGAGGAAGAAGAUGGGC	:.::.:::::: :::::::..::	GUCCGUCUUCUUCCUCUCUUUCG	Cleavage	cDNA|retrotransposon protein, putative, unclassified, expressed	1
GGAGGGAGAGGAAGAAGATGGGC	LOC_Os06g37050.1	3	-1	1	23	165	187	GGAGGGAGAGGAAGAAGAUGGGC	:.::::::::: ::::: ::.::	GUCCAUCUUCUUCCUCCCCUUCG	Cleavage	cDNA|transposon protein, putative, unclassified, expressed	1
GGAGGGAGAGGAAGAAGATGGGC	LOC_Os06g37090.1	3	-1	1	23	165	187	GGAGGGAGAGGAAGAAGAUGGGC	:.::::::::: ::::: ::.::	GUCCAUCUUCUUCCUCCCCUUCG	Cleavage	cDNA|transposon protein, putative, unclassified, expressed	1

GGAGGGAGA GGAAGAAGAT GGGC	LOC_Os 06g3861 2.1	3	-1	1	23	165	187	GGAGGGAGAG GAAGAAGAUG GGC	:.:::::::: ::::: ::.::	GUCCAUCUUC UUCCUCCCCU UCG	Clea vage	cDNA|retrotranspo son protein, putative, unclassified, expressed	1
GGAGGGAGA GGAAGAAGAT GGGC	LOC_Os 06g3862 0.1	3	-1	1	23	165	187	GGAGGGAGAG GAAGAAGAUG GGC	:.:::::::: ::::: ::.::	GUCCAUCUUC UUCCUCCCCU UCG	Clea vage	cDNA|retrotranspo son protein, putative, unclassified, expressed	1
GGAGGGAGA GGAAGAAGAT GGGC	LOC_Os 06g3917 0.1	2.5	-1	1	23	165	187	GGAGGGAGAG GAAGAAGAUG GGC	:.::.::::: :::::::..::	GUCCGUCUUC UUCCUCUCUU UCG	Clea vage	cDNA|retrotranspo son protein, putative, unclassified, expressed	1
GGAGGGAGA GGAAGAAGAT GGGC	LOC_Os 06g3994 0.1	3	-1	1	23	165	187	GGAGGGAGAG GAAGAAGAUG GGC	:.:::::::: ::::: ::.::	GUCCAUCUUC UUCCUCCCCU UCG	Clea vage	cDNA|transposon protein, putative, unclassified, expressed	1
GGAGGGAGA GGAAGAAGAT GGGC	LOC_Os 06g4260 0.1	2.5	-1	1	23	165	187	GGAGGGAGAG GAAGAAGAUG GGC	:.::.::::: :::::::..::	GUCCGUCUUC UUCCUCUCUU UCG	Clea vage	cDNA|retrotranspo son protein, putative, unclassified, expressed	1
GGAGGGAGA GGAAGAAGAT	LOC_Os 06g4911	2	-1	1	23	21	43	GGAGGGAGAG GAAGAAGAUG	:.:::::: :::.:::.:.:	UCUCAUCUUC UUCUUCUUCU	Clea vage	cDNA|delta- aminolevulinic	1

GGGC	0.2							GGC	:.	UCU		acid dehydratase, chloroplast precursor, putative, expressed	
GGAGGGAGA<br>GGAAGAAGAT<br>GGGC	LOC_Os<br>06g4911<br>0.3	2	-1	1	23	5	27	GGAGGGAGAG<br>GAAGAAGAUG<br>GGC	:.:::::::<br>:::.:::.:.:<br>:.	UCUCAUCUUC<br>UUCUUCUUCU<br>UCU	Clea<br>vage	cDNA|delta-aminolevulinic acid dehydratase, chloroplast precursor, putative, expressed	1
GGAGGGAGA<br>GGAAGAAGAT<br>GGGC	LOC_Os<br>06g5080<br>0.1	3	-1	1	23	165	187	GGAGGGAGAG<br>GAAGAAGAUG<br>GGC	:.::::::::<br>::::: ::.::	GUCCAUCUUC<br>UUCCUCCCCU<br>UCG	Clea<br>vage	cDNA|transposon protein, putative, unclassified, expressed	1
GGAGGGAGA<br>GGAAGAAGAT<br>GGGC	LOC_Os<br>07g0165<br>0.1	3	-1	1	23	165	187	GGAGGGAGAG<br>GAAGAAGAUG<br>GGC	:.::::::::<br>::::: ::.::	GUCCAUCUUC<br>UUCCUCCCCU<br>UCG	Clea<br>vage	cDNA|transposon protein, putative, unclassified, expressed	1
GGAGGGAGA<br>GGAAGAAGAT<br>GGGC	LOC_Os<br>07g0923<br>0.1	2.5	-1	1	23	165	187	GGAGGGAGAG<br>GAAGAAGAUG<br>GGC	:.::.::::::<br>:::::::..::	GUCCGUCUUC<br>UUCCUCUCUU<br>UCG	Clea<br>vage	cDNA|retrotransposon protein, putative, unclassified, expressed	1
GGAGGGAGA<br>GGAAGAAGAT<br>GGGC	LOC_Os<br>07g0969<br>0.1	2	-1	1	23	53	75	GGAGGGAGAG<br>GAAGAAGAUG<br>GGC	::.: :::::<br>::::::.:.::<br>:	GCUCCUCUUC<br>UUCCUCUUCU<br>UCC	Clea<br>vage	cDNA|galactosyltransferase family protein, putative,	1

											expressed	
GGAGGGAGA GGAAGAAGAT GGGC	LOC_Os 07g2854 0.1	2.5	-1	1	23	165	187	GGAGGGAGAG GAAGAAGAUG GGC	:.::.::::: :::::::..::	GUCCGUCUUC UUCCUCUCUU UCG	Clea vage	cDNA|retrotranspo son protein, putative, unclassified, expressed	1
GGAGGGAGA GGAAGAAGAT GGGC	LOC_Os 07g3089 0.1	3	-1	1	23	165	187	GGAGGGAGAG GAAGAAGAUG GGC	:.::::::::: ::::: ::.::	GUCCAUCUUC UUCCUCCCCU UCG	Clea vage	cDNA|transposon protein, putative, unclassified, expressed	1
GGAGGGAGA GGAAGAAGAT GGGC	LOC_Os 07g3226 0.1	2.5	-1	1	23	165	187	GGAGGGAGAG GAAGAAGAUG GGC	:.::.::::: :::::::..::	GUCCGUCUUC UUCCUCUCUU UCG	Clea vage	cDNA|retrotranspo son protein, putative, unclassified, expressed	1
GGAGGGAGA GGAAGAAGAT GGGC	LOC_Os 07g3232 0.1	2.5	-1	1	23	165	187	GGAGGGAGAG GAAGAAGAUG GGC	:.::.::::: :::::::..::	GUCCGUCUUC UUCCUCUCUU UCG	Clea vage	cDNA|retrotranspo son protein, putative, unclassified, expressed	1
GGAGGGAGA GGAAGAAGAT GGGC	LOC_Os 07g3666 0.1	2.5	-1	1	23	165	187	GGAGGGAGAG GAAGAAGAUG GGC	:.::.::::: :::::::..::	GUCCGUCUUC UUCCUCUCUU UCG	Clea vage	cDNA|retrotranspo son protein, putative, unclassified, expressed	1
GGAGGGAGA	LOC_Os	3	-1	1	23	165	187	GGAGGGAGAG	:.:::::::::	GUCCAUCUUC	Clea	cDNA|transposon	1

GGAAGAAGAT<br>GGGC	07g3719<br>0.1							GAAGAAGAUG<br>GGC	::::: ::.::	UUCCUCCCCU<br>UCG	vage	protein, putative, unclassified, expressed	
GGAGGGAGA<br>GGAAGAAGAT<br>GGGC	LOC_Os<br>07g4187<br>0.1	3	-1	1	23	165	187	GGAGGGAGAG<br>GAAGAAGAUG<br>GGC	:.::::::::<br>::::: ::.::	GUCCAUCUUC<br>UUCCUCCCCU<br>UCG	Clea<br>vage	cDNA|retrotranspo son protein, putative, unclassified	1
GGAGGGAGA<br>GGAAGAAGAT<br>GGGC	LOC_Os<br>07g4205<br>0.1	3	-1	1	23	165	187	GGAGGGAGAG<br>GAAGAAGAUG<br>GGC	:.::::::::<br>::::: ::.::	GUCCAUCUUC<br>UUCCUCCCCU<br>UCG	Clea<br>vage	cDNA|retrotranspo son protein, putative, unclassified, expressed	1
GGAGGGAGA<br>GGAAGAAGAT<br>GGGC	LOC_Os<br>08g0139<br>0.1	2	-1	1	23	182	204	GGAGGGAGAG<br>GAAGAAGAUG<br>GGC	::.:::::<br>:::.:::.:.:<br>:.	UCCUAUCUUC<br>UUCUUCUUCU<br>UCU	Clea<br>vage	cDNA|phosphatidy linositol-4-phosphate 5-Kinase, putative, expressed	1
GGAGGGAGA<br>GGAAGAAGAT<br>GGGC	LOC_Os<br>08g0152<br>0.1	1.5	-1	1	23	51	73	GGAGGGAGAG<br>GAAGAAGAUG<br>GGC	::.::::<br>::::.:::.::<br>:::	AACCGUCUUC<br>UUCUUCUUCC<br>UCC	Clea<br>vage	cDNA|cytochrome P450, putative, expressed	1
GGAGGGAGA<br>GGAAGAAGAT<br>GGGC	LOC_Os<br>08g0658<br>0.1	2.5	-1	1	23	165	187	GGAGGGAGAG<br>GAAGAAGAUG<br>GGC	:.::.:::::<br>::::::..::	GUCCGUCUUC<br>UUCCUCUCUU<br>UCG	Clea<br>vage	cDNA|retrotranspo son protein, putative, unclassified, expressed	1
GGAGGGAGA	LOC_Os	2.5	-1	1	23	1907	192	GGAGGGAGAG	::.:::::::	GCUCAUCUUC	Clea	cDNA|ABC-2 type	2

GGAAGAAGAT<br>GGGC	08g0701<br>0.1						9	GAAGAAGAUG<br>GGC	::.::::: ::<br>.	UUCUUCUCCG<br>UCU	vage	transporter domain containing protein, expressed	
GGAGGGAGA<br>GGAAGAAGAT<br>GGGC	LOC_Os<br>08g0701<br>0.1	3	-1	1	23	412	434	GGAGGGAGAG<br>GAAGAAGAUG<br>GGC	..: :::::<br>:::.:::.:.:<br>:.	CUUCUUCUUC<br>UUCUUCUUCU<br>UCU	Clea<br>vage	cDNA|ABC-2 type transporter domain containing protein, expressed	2
GGAGGGAGA<br>GGAAGAAGAT<br>GGGC	LOC_Os<br>08g0714<br>0.1	3	-1	1	23	165	187	GGAGGGAGAG<br>GAAGAAGAUG<br>GGC	:.::::::::<br>::::: ::.::	GUCCAUCUUC<br>UUCCUCCCCU<br>UCG	Clea<br>vage	cDNA|transposon protein, putative, unclassified, expressed	1
GGAGGGAGA<br>GGAAGAAGAT<br>GGGC	LOC_Os<br>08g0741<br>0.1	3	-1	1	23	165	187	GGAGGGAGAG<br>GAAGAAGAUG<br>GGC	:.::::::::<br>::::: ::.::	GUCCAUCUUC<br>UUCCUCCCCU<br>UCG	Clea<br>vage	cDNA|retrotransposon protein, putative, unclassified, expressed	1
GGAGGGAGA<br>GGAAGAAGAT<br>GGGC	LOC_Os<br>08g0949<br>0.1	2.5	-1	1	23	193	215	GGAGGGAGAG<br>GAAGAAGAUG<br>GGC	:.::.:::::<br>:::::::..::	GUCCGUCUUC<br>UUCCUCUCUU<br>UCG	Clea<br>vage	cDNA|retrotransposon protein, putative, unclassified	1
GGAGGGAGA<br>GGAAGAAGAT<br>GGGC	LOC_Os<br>08g1448<br>0.1	3	-1	1	23	165	187	GGAGGGAGAG<br>GAAGAAGAUG<br>GGC	:.::::::::<br>::::: ::.::	GUCCAUCUUC<br>UUCCUCCCCU<br>UCG	Clea<br>vage	cDNA|transposon protein, putative, unclassified, expressed	1
GGAGGGAGA<br>GGAAGAAGAT	LOC_Os<br>08g1895	3	-1	1	23	165	187	GGAGGGAGAG<br>GAAGAAGAUG	:.::::::::<br>::::: ::.::	GUCCAUCUUC<br>UUCCUCCCCU	Clea<br>vage	cDNA|transposon protein, putative,	1

GGGC	0.1							GGC		UCG		unclassified, expressed	
GGAGGGAGA GGAAGAAGAT GGGC	LOC_Os 08g1936 0.1	3	-1	1	23	165	187	GGAGGGAGAG GAAGAAGAUG GGC	:.:::::::: ::::: ::.::	GUCCAUCUUC UUCCUCCCCU UCG	Clea vage	cDNA|retrotranspo son protein, putative, unclassified, expressed	1
GGAGGGAGA GGAAGAAGAT GGGC	LOC_Os 08g2028 0.1	2.5	-1	1	23	165	187	GGAGGGAGAG GAAGAAGAUG GGC	:.::.:::::: ::::.:::.::	GUCCGUCUUC UUCCUUUCCU UCG	Clea vage	cDNA|retrotranspo son protein, putative, unclassified, expressed	1
GGAGGGAGA GGAAGAAGAT GGGC	LOC_Os 08g2123 0.1	3	-1	1	23	165	187	GGAGGGAGAG GAAGAAGAUG GGC	:.:::::::: ::::: ::.::	GUCCAUCUUC UUCCUCCCCU UCG	Clea vage	cDNA|retrotranspo son protein, putative, unclassified, expressed	1
GGAGGGAGA GGAAGAAGAT GGGC	LOC_Os 08g2330 0.1	3	-1	1	23	165	187	GGAGGGAGAG GAAGAAGAUG GGC	:.:::::::: ::::: ::.::	GUCCAUCUUC UUCCUCCCCU UCG	Clea vage	cDNA|transposon protein, putative, unclassified, expressed	1
GGAGGGAGA GGAAGAAGAT GGGC	LOC_Os 08g2376 0.1	3	-1	1	23	165	187	GGAGGGAGAG GAAGAAGAUG GGC	:.:::::::: ::::: ::.::	GUCCAUCUUC UUCCUCCCCU UCG	Clea vage	cDNA|retrotranspo son protein, putative, unclassified, expressed	1

GGAGGGAGAGGAAGAAGATGGGC	LOC_Os08g24030.1	3	-1	1	23	165	187	GGAGGGAGAGGAAGAAGAUGGGC	:.::::::::: ::::: ::.::	GUCCAUCUUCUUCCUCCCCUUCG	Cleavage	cDNA|transposon protein, putative, unclassified	1
GGAGGGAGAGGAAGAAGATGGGC	LOC_Os08g25120.1	3	-1	1	23	165	187	GGAGGGAGAGGAAGAAGAUGGGC	:.::::::::: ::::: ::.::	GUCCAUCUUCUUCCUCCCCUUCG	Cleavage	cDNA|transposon protein, putative, unclassified	1
GGAGGGAGAGGAAGAAGATGGGC	LOC_Os08g31010.1	1.5	-1	1	23	1072	1094	GGAGGGAGAGGAAGAAGAUGGGC	::..::: ::: ::::::.:::: :	GCUUAUCCUCUUCCUCUUCCUCC	Cleavage	cDNA|retrotransposon protein, putative, unclassified, expressed	1
GGAGGGAGAGGAAGAAGATGGGC	LOC_Os08g32770.1	3	-1	1	23	165	187	GGAGGGAGAGGAAGAAGAUGGGC	:.::::::::: ::::: ::.::	GUCCAUCUUCUUCCUCCCCUUCG	Cleavage	cDNA|retrotransposon protein, putative, unclassified, expressed	1
GGAGGGAGAGGAAGAAGATGGGC	LOC_Os08g35800.1	2.5	-1	1	23	165	187	GGAGGGAGAGGAAGAAGAUGGGC	:.::.:::::: :::::::..::	GUCCGUCUUCUUCCUCUCUUUCG	Cleavage	cDNA|retrotransposon protein, putative, unclassified, expressed	1
GGAGGGAGAGGAAGAAGATGGGC	LOC_Os08g37550.1	2.5	-1	1	23	165	187	GGAGGGAGAGGAAGAAGAUGGGC	:.::.:::::: :::::::..::	GUCCGUCUUCUUCCUCUCUUUCG	Cleavage	cDNA|retrotransposon protein, putative, unclassified, expressed	1

GGAGGGAGAGGAAGAAGATGGGC	LOC_Os08g40980.1	3	-1	1	23	165	187	GGAGGGAGAGGAAGAAGAUGGGC	:.::.::::::<br>:.:::::..::	GUCCGUCUUCUUUCUCUCUUUCG	Cleavage	cDNA|retrotransposon protein, putative, unclassified, expressed	1
GGAGGGAGAGGAAGAAGATGGGC	LOC_Os08g42640.1	2	-1	1	23	13	35	GGAGGGAGAGGAAGAAGAUGGGC	.::::::::<br>::: :::::::<br>.:	CUCCAUCUUCUUCAUCUCCCUUC	Translation	cDNA|zinc finger, C3HC4 type domain containing protein, expressed	1
GGAGGGAGAGGAAGAAGATGGGC	LOC_Os08g44710.1	3	-1	1	23	165	187	GGAGGGAGAGGAAGAAGAUGGGC	:.::::::::<br>::::: ::.::	GUCCAUCUUCUUCCUCCCCUUCG	Cleavage	cDNA|retrotransposon protein, putative, unclassified	1
GGAGGGAGAGGAAGAAGATGGGC	LOC_Os09g14820.1	3	-1	1	23	165	187	GGAGGGAGAGGAAGAAGAUGGGC	:.::::::::<br>::::: ::.::	GUCCAUCUUCUUCCUCCCCUUCG	Cleavage	cDNA|retrotransposon protein, putative, unclassified, expressed	1
GGAGGGAGAGGAAGAAGATGGGC	LOC_Os09g17380.1	3	-1	1	23	165	187	GGAGGGAGAGGAAGAAGAUGGGC	:.::.::::::<br>:::::::..:.	GUCCGUCUUCUUCCUCUCUUUUG	Cleavage	cDNA|retrotransposon protein, putative, unclassified, expressed	1
GGAGGGAGAGGAAGAAGATGGGC	LOC_Os09g19540.1	3	-1	1	23	165	187	GGAGGGAGAGGAAGAAGAUGGGC	:.::::::::<br>::::: ::.::	GUCCAUCUUCUUCCUCCCCUUCG	Cleavage	cDNA|transposon protein, putative, unclassified,	1

												expressed	
GGAGGGAGAGGAAGAAGATGGGC	LOC_Os09g23800.1	3	-1	1	23	165	187	GGAGGGAGAGGAAGAAGAUGGGC	:.:::::::::::::: ::.::	GUCCAUCUUCUUCCUCCCCUUCG	Cleavage	cDNA|retrotransposon protein, putative, unclassified	1
GGAGGGAGAGGAAGAAGATGGGC	LOC_Os09g24760.1	2	-1	1	23	165	187	GGAGGGAGAGGAAGAAGAUGGGC	:.::::::::::::::::..::	GUCCAUCUUCUUCCUCUCUUUCG	Cleavage	cDNA|retrotransposon protein, putative, unclassified, expressed	1
GGAGGGAGAGGAAGAAGATGGGC	LOC_Os09g25110.1	3	-1	1	23	165	187	GGAGGGAGAGGAAGAAGAUGGGC	:.:::::::::::::: ::.::	GUCCAUCUUCUUCCUCCCCUUCG	Cleavage	cDNA|transposon protein, putative, unclassified	1
GGAGGGAGAGGAAGAAGATGGGC	LOC_Os09g27260.1	2	-1	1	23	50	72	GGAGGGAGAGGAAGAAGAUGGGC	:.:.:::::::.:::.:.:::	CCUCGUCUUCUUCUUCUUCUUCC	Cleavage	cDNA|plant viral response family protein, putative, expressed	1
GGAGGGAGAGGAAGAAGATGGGC	LOC_Os10g08910.1	3	-1	1	23	165	187	GGAGGGAGAGGAAGAAGAUGGGC	:.::.::::::::::::..:.	GUCCGUCUUCUUCCUCUCUUUUG	Cleavage	cDNA|retrotransposon protein, putative, unclassified, expressed	1
GGAGGGAGAGGAAGAAGATGGGC	LOC_Os10g16850.1	3	-1	1	23	165	187	GGAGGGAGAGGAAGAAGAUGGGC	:.::.::::::::::::..:.	GUCCGUCUUCUUCCUCUCUUUUG	Cleavage	cDNA|retrotransposon protein, putative, unclassified,	1

												expressed	
GGAGGGAGAGGAAGAAGATGGGC	LOC_Os10g22540.1	2.5	-1	1	23	165	187	GGAGGGAGAGGAAGAAGAUGGGC	:.::.:::::::::::::..::	GUCCGUCUUCUUCCUCUCUUUCG	Cleavage	cDNA|retrotransposon protein, putative, unclassified, expressed	1
GGAGGGAGAGGAAGAAGATGGGC	LOC_Os10g28460.1	2.5	-1	1	23	165	187	GGAGGGAGAGGAAGAAGAUGGGC	:.::.:::::::::::::..::	GUCCGUCUUCUUCCUCUCUUUCG	Cleavage	cDNA|retrotransposon protein, putative, unclassified, expressed	1
GGAGGGAGAGGAAGAAGATGGGC	LOC_Os10g30600.1	1	-1	1	23	29	51	GGAGGGAGAGGAAGAAGAUGGGC	.:::::::::::::::.:::.	CAUCAUCUUCUUCCUCUCUCUCU	Cleavage	cDNA|tyrosine protein kinase domain containing protein, putative, expressed	1
GGAGGGAGAGGAAGAAGATGGGC	LOC_Os10g30600.2	1	-1	1	23	14	36	GGAGGGAGAGGAAGAAGAUGGGC	.:::::::::::::::.:::.	CAUCAUCUUCUUCCUCUCUCUCU	Cleavage	cDNA|tyrosine protein kinase domain containing protein, putative, expressed	1
GGAGGGAGAGGAAGAAGATGGGC	LOC_Os10g36440.1	2.5	-1	1	23	165	187	GGAGGGAGAGGAAGAAGAUGGGC	:.::.:::::::::::::..::	GUCCGUCUUCUUCCUCUCUUUCG	Cleavage	cDNA|retrotransposon protein, putative, unclassified, expressed	1

GGAGGGAGAGGAAGAAGATGGGC	LOC_Os10g41250.1	1.5	-1	1	23	211	233	GGAGGGAGAGGAAGAAGAUGGGC	..:.:::::::::.:::.::::::	CUUCGUCUUCUUCUUCUUCCUCC	Cleavage	cDNA|glycoprotein, putative, expressed	1
GGAGGGAGAGGAAGAAGATGGGC	LOC_Os11g01510.1	3	-1	1	23	14	36	GGAGGGAGAGGAAGAAGAUGGGC	:::: ::::::::.:::.:.::.	GCCCUUCUUCUUCUUCUUCUUCU	Cleavage	cDNA|ubiquitin-activating enzyme, putative, expressed	1
GGAGGGAGAGGAAGAAGATGGGC	LOC_Os11g01510.2	3	-1	1	23	13	35	GGAGGGAGAGGAAGAAGAUGGGC	:::: ::::::::.:::.:.::.	GCCCUUCUUCUUCUUCUUCUUCU	Cleavage	cDNA|ubiquitin-activating enzyme, putative, expressed	1
GGAGGGAGAGGAAGAAGATGGGC	LOC_Os11g01510.3	3	-1	1	23	2	24	GGAGGGAGAGGAAGAAGAUGGGC	:::: ::::::::.:::.:.::.	GCCCUUCUUCUUCUUCUUCUUCU	Cleavage	cDNA|ubiquitin-activating enzyme, putative, expressed	1
GGAGGGAGAGGAAGAAGATGGGC	LOC_Os11g12700.1	3	-1	1	23	165	187	GGAGGGAGAGGAAGAAGAUGGGC	:.::::::::::::: ::.::	GUCCAUCUUCUUCCUCCCCUUCG	Cleavage	cDNA|retrotransposon protein, putative, unclassified, expressed	1
GGAGGGAGAGGAAGAAGATGGGC	LOC_Os11g28130.1	2	-1	1	23	35	57	GGAGGGAGAGGAAGAAGAUGGGC	:.:::::::.::::::::::	CCUCAUCUUCCUUCUCUCCCUCC	Cleavage	cDNA|expressed protein	2
GGAGGGAGAGGAAGAAGATGGGC	LOC_Os11g28130.1	1.5	-1	1	21	1	21	GGAGGGAGAGGAAGAAGAUGG	.::::::::.::::::::	UCAUCUUCUUUCUCUCCCUCA	Cleavage	cDNA|expressed protein	2
GGAGGGAGAGGAAGAAGATGGGC	LOC_Os11g32060.1	1.5	-1	1	23	1072	1094	GGAGGGAGAGGAAGAAGAUGGGC	::..::: :::::::::.:::::	GCUUAUCCUCUUCCUCUUCCUCC	Cleavage	cDNA|retrotransposon protein, putative,	1

												unclassified, expressed	
GGAGGGAGA GGAAGAAGAT GGGC	LOC_Os 11g3442 0.1	2.5	-1	1	23	165	187	GGAGGGAGAG GAAGAAGAUG GGC	:.::.:::::: :::::::..::	GUCCGUCUUC UUCCUCUCUU UCG	Clea vage	cDNA|retrotranspo son protein, putative, Ty3-gypsy subclass, expressed	1
GGAGGGAGA GGAAGAAGAT GGGC	LOC_Os 11g3454 0.1	2.5	-1	1	23	165	187	GGAGGGAGAG GAAGAAGAUG GGC	:.::.:::::: :::::::..::	GUCCGUCUUC UUCCUCUCUU UCG	Clea vage	cDNA|retrotranspo son protein, putative, unclassified, expressed	1
GGAGGGAGA GGAAGAAGAT GGGC	LOC_Os 11g3459 0.1	2.5	-1	1	23	165	187	GGAGGGAGAG GAAGAAGAUG GGC	:.::.:::::: :::::::..::	GUCCGUCUUC UUCCUCUCUU UCG	Clea vage	cDNA|retrotranspo son protein, putative, unclassified, expressed	1
GGAGGGAGA GGAAGAAGAT GGGC	LOC_Os 11g4213 0.1	3	-1	1	23	165	187	GGAGGGAGAG GAAGAAGAUG GGC	:.:::::::: ::::: ::.::	GUCCAUCUUC UUCCUCCCCU UCG	Clea vage	cDNA|transposon protein, putative, unclassified	1
GGAGGGAGA GGAAGAAGAT GGGC	LOC_Os 11g4568 0.1	3	-1	1	23	165	187	GGAGGGAGAG GAAGAAGAUG GGC	:.:::::::: ::::: ::.::	GUCCAUCUUC UUCCUCCCCU UCG	Clea vage	cDNA|transposon protein, putative, unclassified, expressed	1
GGAGGGAGA GGAAGAAGAT	LOC_Os 11g4720	3	-1	1	23	165	187	GGAGGGAGAG GAAGAAGAUG	:.:::::::: ::::: ::.::	GUCCAUCUUC UUCCUCCCCU	Clea vage	cDNA|transposon protein, putative,	1

GGGC	0.1							GGC		UCG		unclassified, expressed	
GGAGGGAGA GGAAGAAGAT GGGC	LOC_Os 12g0985 0.1	2.5	-1	1	23	165	187	GGAGGGAGAG GAAGAAGAUG GGC	:.::.::::: :::::::..::	GUCCGUCUUC UUCCUCUCUU UCG	Clea vage	cDNA|retrotranspo son protein, putative, Ty3-gypsy subclass, expressed	1
GGAGGGAGA GGAAGAAGAT GGGC	LOC_Os 12g1215 0.1	3	-1	1	23	165	187	GGAGGGAGAG GAAGAAGAUG GGC	:.::::::::: ::::: ::.::	GUCCAUCUUC UUCCUCCCCU UCG	Clea vage	cDNA|transposon protein, putative, unclassified, expressed	1
GGAGGGAGA GGAAGAAGAT GGGC	LOC_Os 12g1569 0.1	2.5	-1	1	23	165	187	GGAGGGAGAG GAAGAAGAUG GGC	:.::.::::: :::::::..::	GUCCGUCUUC UUCCUCUCUU UCG	Clea vage	cDNA|retrotranspo son protein, putative, unclassified, expressed	1
GGAGGGAGA GGAAGAAGAT GGGC	LOC_Os 12g2172 0.1	2.5	-1	1	23	430	452	GGAGGGAGAG GAAGAAGAUG GGC	:.::.::::: :::::::..::	GUCCGUCUUC UUCCUCUCUU UCG	Clea vage	cDNA|retrotranspo son protein, putative, unclassified, expressed	1
GGAGGGAGA GGAAGAAGAT GGGC	LOC_Os 12g3035 0.1	3	-1	1	23	165	187	GGAGGGAGAG GAAGAAGAUG GGC	:.::::::::: ::::: ::.::	GUCCAUCUUC UUCCUCCCCU UCG	Clea vage	cDNA|transposon protein, putative, unclassified	1
GGAGGGAGA GGAAGAAGAT	LOC_Os 12g3656	2.5	-1	1	23	165	187	GGAGGGAGAG GAAGAAGAUG	:.::.::::: :::::::..::	GUCCGUCUUC UUCCUCUCUU	Clea vage	cDNA|retrotranspo son protein,	1

GGGC	0.1							GGC		UCG		putative,<br>unclassified,<br>expressed	
GGAGGGAGA<br>GGAAGAAGAT<br>GGGC	LOC_Os<br>12g3700<br>0.1	3	-1	1	23	165	187	GGAGGGAGAG<br>GAAGAAGAUG<br>GGC	:.::::::::<br>::::: ::.::	GUCCAUCUUC<br>UUCCUCCCCU<br>UCG	Clea<br>vage	cDNA|retrotranspo<br>son protein,<br>putative,<br>unclassified	1
GGAGGGAGA<br>GGAAGAAGAT<br>GGGC	LOC_Os<br>12g3745<br>0.1	3	-1	1	23	165	187	GGAGGGAGAG<br>GAAGAAGAUG<br>GGC	:.::::::::<br>::::: ::.::	GUCCAUCUUC<br>UUCCUCCCCU<br>UCG	Clea<br>vage	cDNA|transposon<br>protein, putative,<br>unclassified,<br>expressed	1
GGAGGGAGA<br>GGAAGAAGAT<br>GGGC	LOC_Os<br>12g4067<br>0.1	2.5	-1	1	23	165	187	GGAGGGAGAG<br>GAAGAAGAUG<br>GGC	:.::.::::::<br>:::::::..::	GUCCGUCUUC<br>UUCCUCUCUU<br>UCG	Clea<br>vage	cDNA|retrotranspo<br>son protein,<br>putative,<br>unclassified,<br>expressed	1
GGAGGGAGA<br>GGAAGAAGAT<br>GGGC	LOC_Os<br>12g4269<br>0.1	2.5	-1	1	23	165	187	GGAGGGAGAG<br>GAAGAAGAUG<br>GGC	:.::.::::::<br>:::::::..::	GUCCGUCUUC<br>UUCCUCUCUU<br>UCG	Clea<br>vage	cDNA|retrotranspo<br>son protein,<br>putative,<br>unclassified,<br>expressed	1
GGAGGGAGA<br>GGAAGAAGAT<br>GGGC	LOC_Os<br>12g4436<br>0.1	1	-1	1	23	453	475	GGAGGGAGAG<br>GAAGAAGAUG<br>GGC	:::::<br>:::::.:::.:<br>::::	CUGCAUCUUC<br>UUCUUCUUCC<br>UCC	Clea<br>vage	cDNA|sodium/hyd<br>rogen exchanger 7,<br>putative, expressed	1
GGAGGGAGA<br>GGAAGAAGAT	LOC_Os<br>12g4436	1	-1	1	23	118	140	GGAGGGAGAG<br>GAAGAAGAUG	:::::<br>:::::.:::.:	CUGCAUCUUC<br>UUCUUCUUCC	Clea<br>vage	cDNA|sodium/hyd<br>rogen exchanger 7,	1

GGGC	0.2							GGC	::::	UCC		putative, expressed	
GGAGGGAGA	LOC_Os	1	-1	1	23	118	140	GGAGGGAGAG	:::::	CUGCAUCUUC	Clea	cDNA|sodium/hyd	1
GGAAGAAGAT	12g4436							GAAGAAGAUG	:::::.:::.:	UUCUUCUUCC	vage	rogen exchanger 7,	
GGGC	0.3							GGC	::::	UCC		putative, expressed	
GGAGGGAGA	LOC_Os	1	-1	1	23	118	140	GGAGGGAGAG	:::::	CUGCAUCUUC	Clea	cDNA|sodium/hyd	1
GGAAGAAGAT	12g4436							GAAGAAGAUG	:::::.:::.:	UUCUUCUUCC	vage	rogen exchanger 7,	
GGGC	0.4							GGC	::::	UCC		putative, expressed	

Most potential targets are labeled red.

Table S2: List of primers used in this article

Primer name	Sequence (5'-3')
Osa-miR11117-Ox F	CCAAGCTTGGTAGACAACTCCTATAACCACC
Osa-miR11117-Ox R	CGGGATCCCTTAGAGTCAGGGTGGTTGTGT
Osa-miR11117-mimic-F	CCAAGCTTGCCCATCTTCTTCCTACTCT
Osa-miR11117-mimic-R	CGGGATCCGGAGGGAGAGTAGGAAGAAG
Osa-miR11117-GUS-F	CGGGATCCCCCTTTACTAGACCAGCTTAGCATAT
Osa-miR11117-GUS-R	CATGCCATGGTCCTCTCATCCTCCTTTTCACTAGTAG
Osa-miR11117-Biotin	GCCCATCTTCTTCCTCTCCCTCC-Biotin
U6-Biotin	TGCGTGTCATCCTTGCGCAGGGGCCATGCT-Biotin
Osa-miR11117-STL	GTCGTATCCAGTGCAGGGTCCGATACGACGCCCATCTGAGGTATTCGCACTG
Osa-miR11117-Foward	GGAGGGAGAGGAAGAAGATGGGC
U6-F for RT	CGATAAAATTGGAACGATACAGA

U6-R for RT	ATTTGGACCATTTCTCGATTTGT
ko-Osa-miR11117-T1-F	TAGGTCTCCCAAAAGTGAGGAGTTTTAGAGCTAGAA
ko-Osa-miR11117-T1-R	CGGGTCTCATTTGTATATTCCTGCACCAGCCGGGAA
ko-Osa-miR11117-T2-F	TAGGTCTCCTATCGACCAAAGGTTTTAGAGCTAGAA
koOsa-miR11117-T2-R	CGGGTCTCAGATAGTCCTATGTGCACCAGCCGGGAA
L5AD5-F	CGGGTCTCAGGCAGGATGGGCAGTCTGGGCAACAAAGCACCAGTGG
L3AD5-R	TAGGTCTCCAAACGGATGAGCGACAGCAAACAAAAAAAAAAGCACCGACTCG
Recom-F	GTGCAGATGATCCGTGGCAACAAAGCACCAGTGGT
Recom-R	CTATTTCTAGCTCTA AAACAAAAAAAAAAGCACCGACTCGGTG
pRGEB32-cx F	TTACGCAATTGGACGACAAC
pRGEB32-cx R	CTTCTGGCTGGTTTGTTGGT
35S-LOC_Os02g41550.3-GFP-F	TTGGAGAGAACACGGGGGACTGCGCCCACCCTCGATCT
35S-LOC_Os02g41550.3-GFP-R	CCTCGCCCTTGCTCACCATGCCCAGGGACCAAATCCCAC
35S-LOC_Os02g57410.10-GFP-F	TTGGAGAGAACACGGGGGACCCACCTCAAAAAAAAAAAAAAAACTCAAGAACAC
35S-LOC_Os02g57410.10-GFP-R	CCTCGCCCTTGCTCACCATGCGATTCCCCGGAATACGATC
35S-LOC_Os04g35890.1-GFP-F	TTGGAGAGAACACGGGGGACATTTCCCCTTCTCCATCTTCT
35S-LOC_Os04g35890.1-GFP-R	CCTCGCCCTTGCTCACCATCCACCACCGCATCGACATT
35S-LOC_Os04g56530.1-GFP-F	TTGGAGAGAACACGGGGGACACGCCCCCCGAAAACAAAATAATTAC
35S-LOC_Os04g56530.1-GFP-R	CCTCGCCCTTGCTCACCATTAACACCACCAGAACACGAATTCC
35S-LOC_Os05g35200.2-GFP-F	TTGGAGAGAACACGGGGGACGGAGAGATACAGAGAGAGGCAGAGGAG

35S-LOC_Os05g35200.2-GFP-R	CCTCGCCCTTGCTCACCATCCCAATGCAAACATGAGCCTGAC
35S-LOC_Os05g51380.2-GFP-F	TTGGAGAGAACACGGGGGACGGCGAGCTCCATGGGGAAG
35S-LOC_Os05g51380.2-GFP-R	CCTCGCCCTTGCTCACCATCGGGGAAGATCCGCTTTGAC
35S-LOC_Os08g01390.1-GFP-F	TTGGAGAGAACACGGGGGACGGAATACACTAGAGTCGGGAAACAGC
35S-LOC_Os08g01390.1-GFP-R	CCTCGCCCTTGCTCACCATCCCAAGTGAAGCAAGAAAAGGAAAC
35SOsPAO4-GFP-F	TTGGAGAGAACACGGGGGACGTTCTTCATCTCGATTCCCATACGT
35SOsPAO4-GFP-R	CCTCGCCCTTGCTCACCATCTCCAGTTTTGAGGCTATTGGGA
35SOsPAO4mts-R1	ACCTTCTTCCTCTTCGTGTTCCAGACAAGCTAGATCTAGC
35SOsPAO4mts-F2	GCTAGATCTAGCTTGTCTGGAACACGAAGAGGAAGAAGGT
Actin-F	GAAGATCACTGCCTTGCTCC
Actin-R	CGATAACAGCTCCTCTTGGC
pOX-OsPAO4 F	GGGTACCGGCGCGCCAAGCTTATGGATCCCAATAGCCTCAAAA
pOX-OsPAO4 R	CAATTCACACTTGTAGGATCCTCAGGTCCTGCAAATCTGAAGG
ko-OsPAO4 F1	TAGGTCTCCTAGCCTCAAAACGTTTTAGAGCTAGAA
ko-OsPAO4 R1	CGGGTCTCAGCTATTGGGATCTGCACCAGCCGGGAA
ko-OsPAO4 F2	TAGGTCTCCGCCCAATTGATAGTTTTAGAGCTAGAA
ko-OsPAO4 R2	CGGGTCTCAGGGCATCCGAAATGCACCAGCCGGGAA
qPCR-OsPAO1 F	GGATCCTCACGTCCGAGTTC
qPCR-OsPAO1 R	CTCGTACGGGAGACCACGA
qPCR-OsPAO2 F	GCCGGTAGACATGGTGGTAG

qPCR-OsPAO2 R	CTCCGTCTTGACGGTGACTC
qPCR-OsPAO3 F	TGAGGAAAATCCCCTGGCAC
qPCR-OsPAO3 R	ACCAGCTCTTGGGGAACTTG
qPCR-OsPAO4 F	TGCTTCGTTTGAGGTGACTG
qPCR-OsPAO4 R	CAATGGTGCCAAGGAGTTCT
qPCR-OsPAO5 F	GTGCGCTGTCTAATGCTTCG
qPCR-OsPAO5 R	CTCCTGGGGAACTTGACGAC
qPCR-OsPAO6 F	CCTACGACTACGAGTTCGCC
qPCR-OsPAO6 R	AGATCTCACGCACCACCTTG
qPCR-OsPAO7 F	AGGGCGTCAATGGTGAGAAG
qPCR-OsPAO7 R	ACTGTCGAAGTCGGAGAGGA
qPCR-OsPR1a F	GGAAGTACGGCGAGAACATC
qPCR-OsPR1a R	GGTCGTACCACTGCTTCTCC
qPCR-OsPR1b F	AGAACTACGCCAGCCAGAGA
qPCR-OsPR1b R	CAGAAGAGGTTCTCGCCAAG
qPCR-OsPR10a F	GCCAAGCTCAAGGTGGAGTA
qPCR-OsPR10a R	AGTAGCCATCCACGATGTCC
qPCR-OsERF3 R	CAAACTTTCCGTTTGCATCA
qPCR-OsERF3 F	CCGGTGTCTACCGTGCTATT
qPCR-OsACO1 F	GACGTTCCCGGTCATCAACA

qPCR-OsACO1 R	ACGCTTGTAGTGGTCCTTGG
qPCR-OsACO2 F	TTGAGATCCTGAACCACGGC
qPCR-OsACO2 R	CACACGCTTGTAGTGGTCCT
qPCR-OsACO3 F	CGACCAGATCGACACGACAA
qPCR-OsACO3 R	TGGTTCAGCATCTGTGCAGT
qPCR-OsACS1 F	ATGGTGAGCCAAGTGGTCG
qPCR-OsACS1 R	CCCCAGGAAGTAGGACGAGT
HygR-F	AAGATGTTGGCGACCTCGTATT
HygR-R	CGTGCTTTCAGCTTCGATGTAG
